# Pathological multifocality is not a prognosis factor of papillary thyroid carcinoma: a single-center, retrospective study

**DOI:** 10.1186/s12957-022-02869-8

**Published:** 2022-12-12

**Authors:** Yoko Omi, Kento Haniu, Hidenori Kamio, Mikiko Fujimoto, Yusaku Yoshida, Kiyomi Horiuchi, Takahiro Okamoto

**Affiliations:** Department of Endocrine Surgery, Tokyo Women’s Medical University, Tokyo, Japan

**Keywords:** Papillary thyroid carcinoma, Multifocal

## Abstract

**Introduction:**

Non-total thyroidectomy (non-TTx) is a widely accepted operative procedure for low-risk papillary thyroid carcinoma (PTC). PTC patients preoperatively diagnosed with unifocal disease are often revealed as having multifocal foci by microscopy. The present study determined whether or not patients with clinically unifocal, but pathologically multifocal non-high-risk PTC treated with non-TTx have an increased risk of a poor prognosis compared to those with pathologically unifocal PTC.

**Materials and methods:**

PTC patients diagnosed as unifocal preoperatively who underwent non-TTx were multifocal in 61 and unifocal in 266 patients microscopically. Oncologic event rates were compared between pathologically multifocal and unifocal PTC patients.

**Results:**

Pathological multifocality was associated with positive clinical lymph node metastasis (cN1) (odds ratio [OR] 4.01, 95% confidence interval [CI]: 1.91–8.04) and positive pathological lymph node metastasis (pN1) in > 5 nodes (OR 3.68, 95% CI: 1.60–8.49). No patients died from PTC. There was no significant difference in the disease-free survival rate, remnant thyroid disease-free survival rate, lymph node disease-free survival rate, or distant disease-free survival rate between the two groups. Recurrence in pathologically multifocal PTC patients was locoregional in all cases and able to be salvaged by reoperation. Cox proportional hazard model analyses showed no significant difference in recurrence rates with regard to pathological multifocality and cN or number of pNs.

**Conclusion:**

The prognosis of PTC with pathological multifocality treated by non-TTx was not inferior to that of unifocal PTC. Immediate completion thyroidectomy is not necessary when microscopic foci are proven.

## Introduction

Papillary thyroid carcinoma (PTC) is the most common endocrine malignancy, and its incidence has been increasing worldwide [1]. Management of PTC has evolved as reflected in professional societies’ clinical practice guidelines [2–5]. The latest Japanese guidelines set four risk categories predictive of recurrence or cancer death as an initial assessment of PTC based on the preoperative findings according to the TNM classification: very low-risk PTC measuring ≤ 1 cm without any metastases (T1aN0M0); low-risk PTC measuring 1.1–2 cm without any metastases (T1bN0M0); high-risk PTC having at least 1 of several features including tumor size > 4 cm, extrathyroidal extension, or extranodal extension to adjacent structures except for the sternothyroid muscle, clinical node metastasis > 3 cm, and M1; and intermediate-risk PTC, referring to a tumor that does not meet any of the definitions for very low-, low-, or high-risk categories [5].

Aside from active surveillance for very low-risk PTC, hemithyroidectomy (lobectomy or lobectomy plus isthmectomy) along with prophylactic central node dissection is a treatment of choice for the very low-risk, the low-risk, and even some intermediate-risk PTCs [5]. Those surgical procedures have the advantages of avoiding postoperative thyroid hormone replacement and hypoparathyroidism; however, the incidental microscopic multifocality of the disease poses a dilemma. Indeed, multifocal PTC accounts for 18–87% of PTCs [6].

In cases of clinically unifocal PTC treated with non-total thyroidectomy (non-TTx), incidental microscopic PTC is often recognized. In such cases, PTC not detected clinically might be left in the remaining thyroid tissue. Multifocality in the ipsilateral lobe is a risk factor of cancer presence in the opposite lobe that the rate accounts for 16.7–58% [7–16]. The European Society of Endocrine Surgeons recommends total or near-total thyroidectomy for apparent multifocal PTC as the initial treatment to reduce local recurrence risk [6]. It also states that completion thyroidectomy may be necessary when the diagnosis of multifocal PTC is made following non-TTx [6]. While the probability of microscopic residual cancer may be high in multifocal PTC treated with non-TTx, the need to add completion surgery remains controversial [6–8, 17].

In the previous reports, the multifocality of cases was not clearly described. How microscopic multifocality influences the outcomes of PTC patients thus remains unclear. To address this uncertainty, we conducted a retrospective survey with a study population of patients with pathologically multifocal PTC and those with pathologically proven unifocal PTC treated by non-TTx. The research questions of this study are as follows: is there a difference in oncologic event rates between pathologically multifocal and unifocal PTC treated by non-TTx? What is the likelihood of developing recurrence in patients with pathologically multifocal PTC relative to those with unifocal PTC after controlling for potential confounders?

## Patients and methods

### Study population

**Fig. 1:**
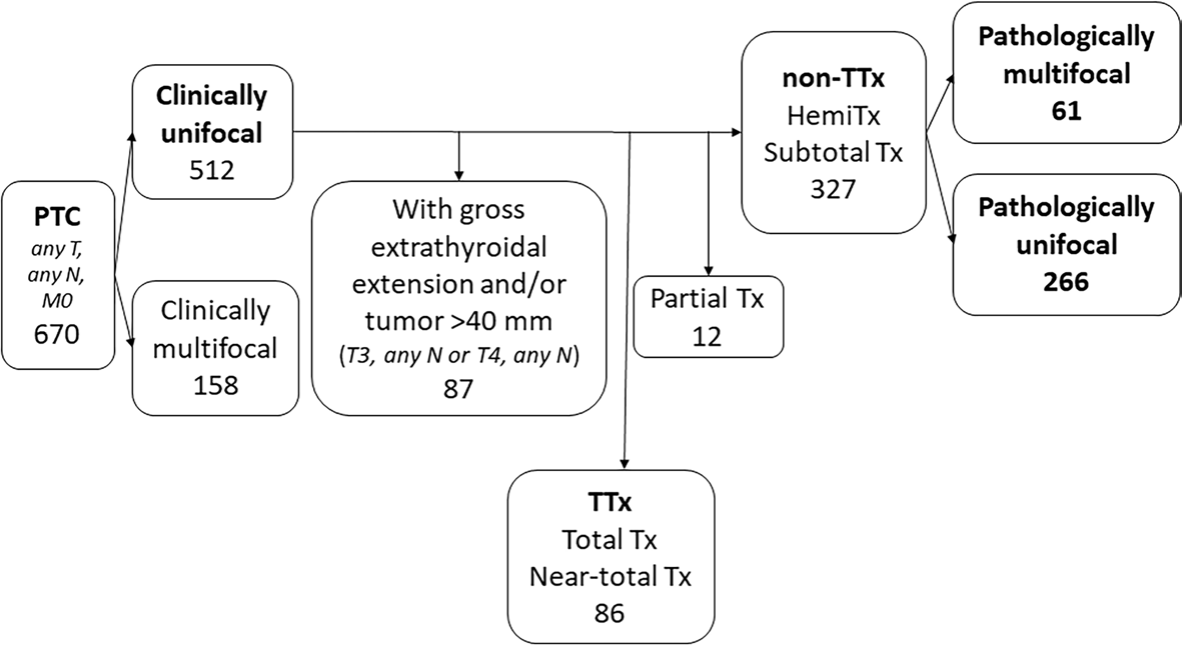
Inclusion and exclusion criteria. Eight edition of AJCC TNM classification for papillary thyroid carcinoma is described in italic

Six-hundred and seventy patients underwent an initial operation for PTC without distant metastasis at Tokyo Women’s Medical University from 2010 to 2017. Clinically, PTC was unifocal in 512 (76.4%) patients. Patients with gross extrathyroidal extension and/or with tumors > 4 cm in size, who underwent total thyroidectomy (TTx) or near TTx, non-TTx followed by completion thyroidectomy (CTx) within 6 months, or who underwent partial thyroidectomy, were excluded. Ultimately, 327 patients who underwent non-TTX, including 320 hemithyroidectomy and 7 subtotal thyroidectomy patients, were enrolled. Serial 5-mm sections of whole specimen were prepared for pathological examination. Pathologically multifocal PTC was found in 61 (18.7%) of the non-TTx patients (Fig. 1). The study included 327 non-high-risk patients who underwent non-TTx for clinically unifocal PTC.

### Observations

We retrospectively reviewed the medical record of each patient to collect data on the following: sex, age at surgery, preoperative and postoperative 8th edition UICC TNM classification, use of levothyroxine replacement, and vital status. Preoperative TNM classification was evaluated by neck ultrasonography and/or computed tomography (CT). Patients were followed every 6 to 12 months by both physical examinations and thyroid function tests including serum thyroglobulin evaluations, in conjunction with neck ultrasonography. CT scans were regularly performed on patients with high-risk features, and they were also carried out as needed for those with either apparent or suspected disease recurrence. The oncologic events that account for the study outcomes were the recurrence of PTC (primary) and cancer death (secondary).

### Statistical analyses

Student’s *t*-test and *χ*^2^ test were used to compare variables between groups. The disease-free survival (DFS) and disease-specific survival were estimated using the Kaplan-Meier method. The log-rank test was used for the univariable analysis. The hazard ratio was calculated by a Cox proportional hazard model. A *p*-value < 0.05 was considered significant. All statistical analyses were performed using the JMP R Pro software program, 14.0.0 (SAS Institute, Cary, NC, USA). This study was approved by the ethics committee of Tokyo Women’s Medical University (reference number 4842), and the requirement to obtain informed consent was waived.

## Results

### Clinicopathologic features (Table 1)

Sex distribution and age were not significantly different between the pathologically multifocal and unifocal PTC groups. Distribution of pathological subtype according to 4th edition of WHO classification [18] did not differ between multifocal and unifocal PTC groups (Table 2). Pathological multifocality was associated with clinically positive lymph node metastasis (cN1) (odds ratio [OR] 4.01, 95% confidence interval [CI]: 1.91–8.04) and positive pathological lymph node metastasis (pN1) in > 5 nodes (OR 3.68, 95% CI: 1.60–8.49). All cN1 patients were pN1. Postoperative levothyroxine replacement was necessary in 7 (11.5%) pathologically multifocal PTC patients and 74 (27.8%) pathologically unifocal PTC patients. In total, 246 patients (75.2%) maintained their thyroid function without levothyroxine.

**Table 1:** Clinicopathologic features of patients with pathologically multifocal or unifocal PTC

	Multifocal (***n*** = 61)	Unifocal (***n*** = 266)	Odds ratio (95% ***CI***)	***p-value***
Sex				
Female	49 (80.3%)	189 (71.1%)		
Male	12 (19.7%)	77 (29.0%)	0.60 (0.30–1.19)	*0.14*
Age				
< 55 years	40 (65.6%)	160 (60.2%)		
≥ 55 years	21 (34.4%)	106 (39.9%)	0.79 (0.44–1.42)	*0.43*
Clinical tumor size				
≤ 20 mm	52 (85.3%)	204 (76.6%)		
> 20 mm	9 (14.8%)	62 (23.4%)	0.57 (0.26–1.21)	*0.14*
Clinical lymph node metastasis				
Negative	46 (75.4%)	246 (92.5%)		
Positive	15 (24.6%)	20 (7.5%)	4.01 (1.91–8.04)	*< 0.001*
Pathological tumor size				
≤ 20 mm	55 (90.2%)	219 (82.3%)		
> 20 mm	6 (9.8%)	47 (17.7%)	0.51 (0.21–1.25)	*0.13*
Pathological lymph node metastasis				
Negative	26 (42.6%)	129 (48.5%)		
Positive	35 (57.4%)	137 (51.5%)	1.27 (0.72–2.22)	*0.41*
Number of pathological lymph node metastasis				
≤ 5	50 (82.0%)	251 (94.4%)		
> 5	11 (18.0%)	15 (5.6%)	3.68 (1.60–8.49)	*0.001*

*PTC* papillary thyroid carcinoma, *CI* confidence interval

**Table 2:** Pathological subtype of papillary carcinoma

	Multifocal (***n*** = 61)	Unifocal (***n*** = 266)
Papillary carcinoma	33 (54.1%)	142 (53.4%)
Papillary microcarcinoma	23 (37.7%)	103 (38.7%)
Follicular variant	4 (6.6%)	17 (6.4%)
Encapsulated variant	0	2 (0.8%)
Oncocytic variant	0	1 (0.4%)
Solid variant	0	1 (0.4%)
Cribriform-morular variant	1 (1.6%)	0

### Postoperative events and the survival

**Fig. 2:**
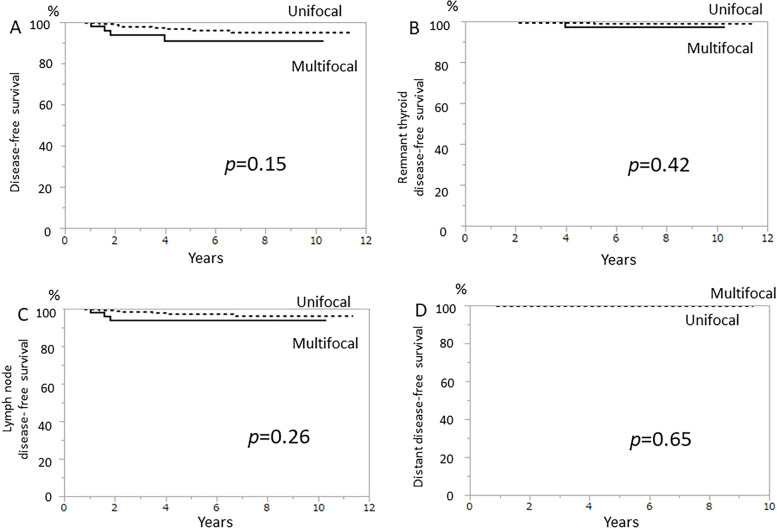
Comparing unifocal and multifocal papillary thyroid carcinoma. Kaplan-Meier curves showing disease-free survival (**A**), remnant thyroid disease-free survival (**B**), lymph node disease-free survival (**C**), distant disease-free survival (**D**)

Median follow-up period was 5.32 years (range: 0.01–11.38). None of the patients died from PTC (Table 3). The 5-year disease-free survival (DFS) rate, remnant thyroid DFS (RDFS), lymph node DFS (LDFS), and distant DFS (DDFS) of pathologically multifocal PTC was 90.9% (95% CI: 82.2–99.6), 97.1% (95% CI: 91.6–100), 94.0% (95% CI: 87.3–100), and 100%, respectively (Fig. 2 A–D). The 5-year DFS, RDFS, LDFS, and DDFS of pathologically unifocal PTC were 96.7% (95% CI: 94.3–99.1), 99.5% (95% CI: 98.6–100), 97.1% (95% CI: 94.7–99.4), and 99.0% (95% CI: 98.5–100), respectively (Fig. 2 A–D). While the DFS of pathologically multifocal PTC was lower than that of pathologically unifocal PTC, there was no significant difference between the 2 groups (*p* = 0.15) (Fig. 2 A). Four patients (6.6%) with pathologically multifocal PTC developed recurrence, 2 in the cervical lymph node, 1 in the mediastinal lymph node, and 1 in the residual thyroid tissue (Table 3). Nine patients (3.4%) with unifocal PTC developed recurrence, 6 in the cervical lymph node only, 1 in the cervical lymph node and lung, and 2 in the residual thyroid tissue (Table 3). Contralateral lobe was resected in 6 patients. PTC in the residual thyroid tissue was detected in 4 (67%) patients. Complication associated with CTx was permanent hypoparathyroidism in one patient and transient recurrent laryngeal nerve palsy in another patient. Cox proportional hazard model analyses showed no significant difference in the hazard ratios of PTC recurrence with regard to pathological multifocality and cN or the number of pNs (Table 4).

**Table 3:** Postoperative events

Postoperative events	Multifocal (***n*** = 61)	Unifocal (***n*** = 269)	***p-value***
Recurrence	4 (6.6%)	9 (3.4%)	*0.21*
Lymph node	3 (4.9%)	7^a^ (2.6%)	*0.28*
Local	0	0	
Distant	0	1^a^ (0.4%)	*1.00*
Remnant thyroid	1 (1.6%)	2 (0.8%)	*0.46*
Death due to PTC	0	0	

*PTC* papillary thyroid carcinoma

^a^Including 1 case where metastases were found in the lymph node and lung at the same time

**Table 4:** Results of Cox proportional hazard model analyses

	HR (95% CI)	***p-value***
Pathological multifocality	2.31 (0.63–7.10)	*0.19*
	HR (95% CI)	***p-value***
Pathological multifocality	1.88 (0.48–6.19)	*0.34*
Clinical lymph node metastasis positive	2.29 (0.48–8.16)	*0.27*
	HR (95% CI)	***p-value***
Pathological multifocality	1.83 (0.47–6.00)	*0.36*
Pathological lymph node metastasis > 5	2.75 (0.58–9.74)	*0.18*

*HR* hazard ratio, *CI* confidence interval

## Discussion

Non-TTx has been the favored operative procedure for PTC including node-positive cases in Japan than in other countries especially before adjuvant radioiodine therapy became available in 2010. In addition to the merits of non-TTx, the shortage of institutions that perform radioiodine therapy and the Japanese historical background of disliking for radiation-using modalities have boosted this tendency. Previous findings that the survival of PTC patients is not markedly affected by the extent of surgery has become the basis of allowing non-TTx for non-high-risk PTC cases [19–24].

Performing non-TTx without CTx for multifocal PTC remains controversial [6–8, 17, 25]. In the guideline of the American Thyroid Association published in 2015, CTx is recommended for multifocal PTC treated with non-TTx to achieve complete resection of multifocal disease and to allow for efficient RAI therapy [3]. Pathologically multifocal PTC in the unilateral lobe increases the possibility of clinically undetected PTC in the remnant thyroid [7, 8, 10–16]. Some studies have reported that lymph node metastasis is more often observed in multifocal PTC than in unifocal PTC, although none of them distinguished cN and pN [26–31].

In the present study, multifocality was associated with cN and > 5 lymph nodes involved pN1. cN was adopted as a factor separately from pN as it indicates large-volume, macroscopic clinically apparent metastasis. This implies that multifocality is related to the massive lymphatic flow of cancer cells. Multifocality is reported to be a risk factor of cancer recurrence [26–29, 32–35]. In contrast, some reports described no marked difference in recurrence [17, 30, 36] or the survival based on multifocality [17, 30, 34, 37].

All the previous studies discussed about clinical multifocality. One unique point of our present study is that the target was multifocality that was recognized only microscopically. In our study, the DFS, RDFS, LDFS, and DDFS of pathologically multifocal PTC were not statistically inferior to those that of pathologically unifocal PTC. Cox proportional hazard model analyses revealed that pathological multifocality was not a risk factor for recurrence. Because of the retrospective nature of the present study, some factors may have affected the association between the multifocality of PTC and disease recurrence. Multivariable analyses, however, indicated that the hazard ratios of pathological multifocality were statistically insignificant. The lymph node was the most frequent site of recurrence. Higher rate of cN1 and higher number of pN1 might affect the higher rate of lymph node recurrence in pathologically multifocal PTC. Indeed, cN1 and > 5 lymph nodes involved pN1 are both reported to be risk factors of locoregional lymph node metastasis [38]. A large volume of lymph node metastasis related to multifocality may be an important risk factor of PTC recurrence. In contrast, recurrence in the remnant thyroid gland was infrequent.

Microscopic PTC may exist in the remnant thyroid gland with high probability, but it did not worsen the prognosis of the multifocal PTC patients. Some studies have reported that PTC found in the remaining thyroid at the time of CTx has no impact on recurrence [13, 39]. All the patients who developed recurrence except for one in the unifocal PTC group who developed lung metastasis were able to be salvaged by a second operation. Complications such as postoperative hematoma, recurrent laryngeal nerve injury, or hypoparathyroidism are reported to occur in 5–20% of the patients undergoing CTx [40–42]. The timing of the CTx does not influence the permanent complication rate [42].

Non-TTx is a feasible operative procedure for maintaining the postoperative thyroid function and avoiding hypoparathyroidism. Recurrence in lymph nodes or remnant thyroid occurred more often, but not significantly so, in pathologically multifocal PTC patients than in pathologically unifocal PTC patients who treated with non-TTx. Such cases of recurrence were able to be cured by reoperation performed at the time that recurrence was discovered. Performing with non-TTx treatment is acceptable for non-high-risk incidentally multifocal PTC. CTx can be postponed until the time of the evident recurrence.

Limitations of this study include its short follow-up time and small sample size, which may have masked the differences. Further studies with a longer follow-up and larger sample size are required to confirm these findings.

## Conclusion

The prognosis of PTC with pathological multifocality treated by non-TTx was not inferior to that of unifocal PTC. Non-TTx is a feasible operative procedure for clinically unifocal PTC without high risk regardless of microscopic multifocality. Immediate CTx is not necessary when microscopic foci are proven.

## Data Availability

Data are available from the corresponding author upon reasonable request.

## Abbreviations

CI: Confidence interval
CT: Computed tomography
CTx: Completion thyroidectomy
DDFS: Distant disease-free survival
DFS: Disease-free survival
LDFS: Lymph node disease-free survival
Non-TTx: Non-total thyroidectomy
NS: Not significant
OR: Odds ratio
PTC: Papillary thyroid carcinoma
RDFS: Remnant thyroid disease-free survival
SD: Standard deviation
TTx: Total thyroidectomy
